# Persistent low expression of hZip1 in mucinous carcinomas of the ovary, colon, stomach and lung

**DOI:** 10.1186/s13048-015-0169-8

**Published:** 2015-06-17

**Authors:** Mohamed Mokhtar Desouki, Renty B. Franklin, Leslie C. Costello, Oluwole Fadare

**Affiliations:** Department of Pathology, Microbiology and Immunology, Vanderbilt University Medical Center, Vanderbilt University School of Medicine, 1161 21st Avenue South, MCN, C-2310A, Nashville, TN 37232-2561 USA; Department of Oncology and Diagnostic Sciences and the Greenebaum Cancer Center/Dental School, University of Maryland, Baltimore, MD 21201 USA; Department of Pathology, San Diego Medical Center, University of California San Diego, San Diego, CA 92103 USA

**Keywords:** Zinc transporter, hZip1, Mucinous carcinomas, Ovary, Colon, Stomach, Lung

## Abstract

**Background:**

Mucinous carcinomas from different organs are morphologically similar and might share similarities at the molecular and biochemical levels that may illuminate their pathogenesis and influence management. The factors involved in the pathogenesis of mucinous carcinomas remain unknown; which is likely one contributor to the current dearth of biomarkers for detection. Because zinc changes are implicated in some cancers e.g., prostate; we assessed the possibility of a similar role in mucinous carcinomas.

**Methods:**

The goal of the current work is to study the expression of hZip1 by immunohistochemistry in mucinous carcinomas as compared with non-neoplastic epithelia and conventional carcinomas. Tissue microarray slides containing mucinous carcinomas of the ovary (*n* = 35), colon (*n* = 51), stomach (*n* = 32) and lung (*n* = 21) were used.

**Results:**

hZip1 showed persistent low expression in mucinous compared to ovarian serous carcinomas and normal tissue (*P* < 0.05), colonic adenocarcinoma and normal mucosa (*P* < 0.001), and gastric adenocarcinoma and normal epithelium (*P* < 0.05). hZip1 also showed low expression in pulmonary mucinous carcinomas.

**Conclusions:**

hZip1 is consistently decreased in mucinous carcinomas from a variety of organs. Despite the fact that these preliminary findings are unlikely to be of much diagnostic significance, these findings suggest that hZip1 plays a fundamental role in the carcinogenesis of mucinous tumors.

## Background

Clinical studies and experimental evidence have established that zinc levels are consistently decreased in prostate adenocarcinoma as compared to normal prostate glands. The Zip1 zinc uptake transporter, in addition to other members of the zinc transporter genes, is responsible for the uptake and accumulation of zinc from the circulation in the normal glands against the concentration gradient. The malignant prostatic glands exhibit a down regulation of Zip1, normally a ubiquitously expressed protein, expression that will eliminate the uptake and accumulation of zinc intracellularly [1–4].

In contrast to the aforementioned role of zinc in the prostate, the role of hZip1 expression in adenocarcinomas of other organs has not previously been comprehensively examined. No such conclusive background exists for epithelial ovarian cancer for example which is considered one of the least understood of all major human malignancies [5]. Identification of the most important initial alterations in ovarian cancer may facilitate the development of better methods for early diagnosis, through the discovery of epithelial ovarian cancer biomarkers, and for the development of more optimal therapeutic approaches that target key molecular pathways [6].

There is evidence that relates zinc as a potential tumor suppressor agent and *ZIP1* transporter as a tumor suppressor gene in some epithelial ovarian cancers. Lightman et al. showed a highly significant decrease in the zinc concentration of malignant ovarian tissue as compared to their non-neoplastic counterparts [7]. Bae et al. showed that zinc treatment of OVCAR-3 cells exerts tumor suppressor effects with increased apoptosis [8]. The OVCAR-3 is a human epithelial ovarian cancer cell line which was established from the malignant ascites of a patient with poorly differentiated “papillary adenocarcinoma” of the ovary [9]. A significant correlation between a decrease in blood and scalp-hair zinc levels with an increase in the incidence of ovarian cancer has been reported [7, 10]. These preliminary data make it reasonable to expect that there is a difference in the zinc levels between malignant and non-malignant cells which may be related to alteration of the zinc uptake transporter; presumably Zip1.

In the current classifications of ovarian neoplasms, mucinous tumors are classified as surface epithelial tumors. Primary mucinous tumors of the ovary are classified into benign, borderline, or malignant categories depending on their histopathologic features. The neoplastic cells may be of gastrointestinal type, endocervical type, or mixtures of both cell types that exist in the seromucinous tumors [11] . Primary invasive mucinous carcinomas of the ovary are very uncommon, accounting for only 3-10 % of all ovarian epithelial tumors, and metastatic tumors, most commonly from the gastrointestinal tract (GIT), are more common [12, 13]. Therefore, the possibility that a mucinous tumor in the ovary is metastatic rather than primary ovarian carcinoma always must be entertained. Common primary sites are GIT, especially the large intestine [14], appendix [15, 16], and pancreas [17] and less commonly from stomach [18]. Endocervical adenocarcinomas occasionally spread to the ovary [19].

We hypothesized that mucinous carcinomas, irrespective of different organ site, might share similarities at the molecular and biochemical levels, which are manifested by similar morphology at different anatomical sites. The anatomical site obviously will dictate the surgical approach; however, the molecular and biochemical characteristics might be more useful in the development and composition of adjuvant treatment protocols.

In this report, we show, for the first time, a consistent pattern of low expression of the hZip1 protein in mucinous carcinomas from different organs, including the ovary, colon, stomach and lung. The evidence presented herein indirectly supports the likelihood that *ZIP1* gene is an essential step in the development of mucinous neoplasms.

## Results

### Low expression of hZip1 in ovarian mucinous carcinomas versus high expression in ovarian serous carcinomas

**Fig. 1:**
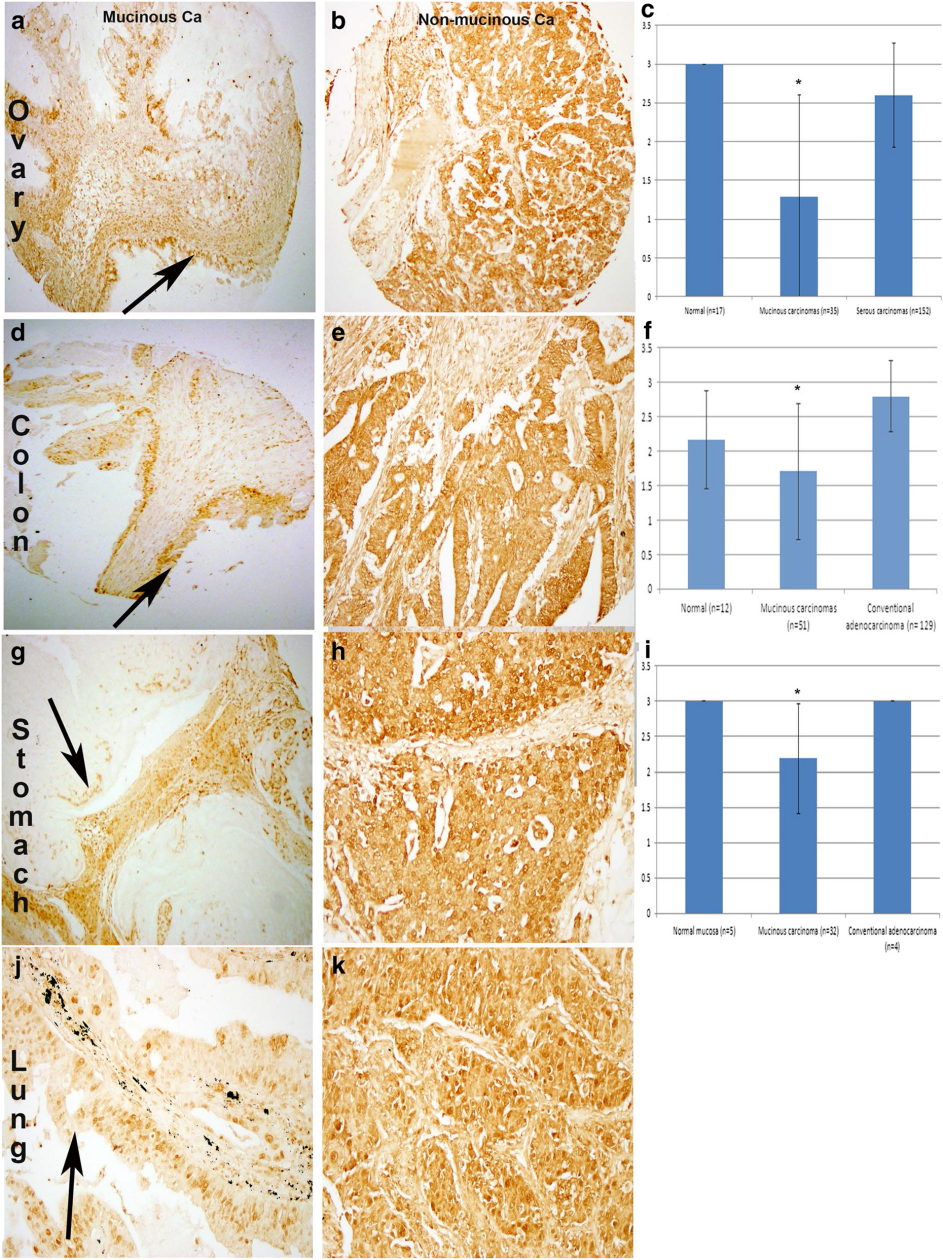
hZip1 expression in mucinous and non-mucinous carcinomas of the ovary, colon, stomach and lung. IHC analysis was performed on tissue microarray slides comprised of 35 ovarian mucinous carcinomas, 152 ovarian serous carcinomas, 51 colonic mucinous carcinomas, 129 conventional colonic adenocarcinomas, 31 gastric mucinous carcinomas, 4 conventional gastric adenocarcinomas and 21 pulmonary mucinous carcinomas. **a** Representative negative ovarian mucinous carcinoma compared to **b** strongly positive serous carcinoma case for hZip1 protein. **c** Diagram showing the average hZip1 IHC scores in normal ovarian, mucinous and serous carcinomas. **d** Representative weak positive colonic mucinous carcinoma compared to **e** strongly positive conventional colonic adenocarcinoma case for hZip1 protein. **f** Diagram showing the average hZip1 IHC scores in normal colonic epithelium, mucinous and conventional colonic adenocarcinomas. **g** Representative negative gastric mucinous carcinoma compared to **h** strongly positive conventional gastric adenocarcinoma case for hZip1 protein. **i** Diagram showing the average hZip1 IHC scores in normal gastric mucosa, mucinous and conventional gastric adenocarcinomas. **j** Representative weak and **k** strong positive pulmonary mucinous carcinoma cases for hZip1 protein. ZIP1 protein was visualized using DAB with hematoxylin counterstain. Arrows showed the negative/weak positive mucinous carcinoma cells. * There is statistically significant difference

The average age of patients with ovarian carcinomas was 51.4 (range of 22–81) years. Tissue microarray (TMA) comprised of 17 normal ovarian tissue, all of which were strongly (3+) positive for hZip1. Sixty percent (21/35) of mucinous carcinomas were negative (0) to weak (1+) positive for hZip1 (Fig. 1a). In contrast, hZip1 expression was moderately (2+) to strongly (3+) positive in 91 % (138/152) of serous carcinomas (Fig. 1b). The expression of hZip1 was significantly lower in ovarian mucinous carcinomas as compared to serous carcinoma (1.3 versus 2.6; P < 0.001) and normal ovarian tissue (P = 0.015) (Fig. 1c).

There was a statistically significant lower expression of hZip1 in low grade serous compared to high grade serous carcinomas (P < 0.05). Also, there was a lower expression of hZip1 in low grade compared to high grade ovarian mucinous carcinomas (P = 0.6). There was no association between clinical stage and hZip1 IHC scores.

### Low expression of hZip1 in mucinous carcinomas versus high expression in conventional adenocarcinoma of the colon

The average age of patients with colonic carcinomas was 57.4 (range of 20–90) years. TMA comprised of 12 normal colonic tissue of which 83 % (10/12) were moderately (2+) to strongly (3+) positive for hZip1. Fifty-five percent (28/51) of mucinous carcinomas were negative (0) to weak (1+) positive for hZip1 (Fig. 1d). In contrast, hZip1 expression was moderately (2+) to strongly (3+) positive in 96 % of conventional colonic adenocarcinoma (124/129) cases (Fig. 1e). The mean expression of hZip1 was significantly lower in colonic mucinous carcinomas (1.7) as compared to colonic conventional adenocarcinomas (2.8) and normal colonic mucosa (2.2) (*P* < 0.001) (Fig. 1f).

hZip1 expression was decreased as a function of increasing grade in both mucinous and non-mucinous tumors of the colon with no statistically significant difference. There was no association between clinical stage and hZip1 IHC scores.

### Low expression of hZip1 in mucinous carcinomas versus high expression in conventional adenocarcinomas of the stomach

The average age of patients with gastric carcinomas was 56 (range of 22–78) years. TMA comprised of 5 normal gastric epithelium all of which were strongly (3+) positive for hZip1. Twenty-two percent (7/32) of mucinous carcinomas were negative (0) to weak (1+) positive for hZip1 (Fig. 1g). In contrast, all conventional adenocarcinoma cases (*n* = 4) were strongly (3+) positive for hZip1 (Fig. 1h). The expression of hZip1 was significantly lower in gastric mucinous carcinomas as compared to conventional gastric adenocarcinoma (2.2 versus 3; *P* < 0.05) and normal gastric epithelium (*P* = 0.027) (Fig. 1i).

The mean score of hZip1 was significantly lower in high grade mucinous carcinomas of the stomach as compared to their low grade counterparts (1.9 vs. 2.7, *p* < 0.01). There was no association between clinical stage and hZip1 IHC scores.

### Low expression of hZip1 in mucinous carcinomas of the lung

The average age of patients with pulmonary mucinous carcinomas was 55.1 (range of 38–71) years. TMA comprised of 21 pulmonary mucinous carcinomas, of which 1, 3, 4 and 13 cases were negative (0), 1+, 2+, and 3+ positive for hZip1 protein expression, respectively with a mean score of 2.4. Normal lung epithelium in adjacent sections strongly (3+) expresses hZip1 (Fig. 1j and k). There was no association between clinical stage and hZip1 IHC scores.

Table 1 summarizes the results of hZip1 protein expression in normal tissue, mucinous and non-mucinous carcinomas of the ovary, colon, stomach and lung.

**Table 1:** hZip1 expression in normal, mucinous and non-mucinous carcinomas of the ovary, colon, stomach and lung

Diagnosis	hZip1 IHC score				Total
	Negative	1+	2+	3+	
Ovary					
Normal	0	0	0	17 (100 %)	17
Mucinous carcinomas	15 (43 %)	6 (17 %)	3 (9 %)	11 (31 %)	35
Serous carcinomas	1 (0.7 %)	13 (9 %)	32 (21 %)	106 (70 %)	152
Colon					
Normal	0	2 (17 %)	6 (50 %)	4 (33 %)	12
Mucinous carcinomas	3 (6 %)	25 (49 %)	7 (14 %)	16 (31 %)	51
Adenocarcinomas	1 (1 %)	4 (3 %)	15 (12 %)	109 (84 %)	129
Stomach					
Normal	0	0	0	5 (100 %)	5
Mucinous carcinomas	0	7 (22 %)	12 (37 %)	13 (41 %)	32
Adenocarcinomas	0	0	0	4 (100 %)	4
Lung					
Normal	0	0	0	21 (100 %)	21
Mucinous carcinomas	1 (5 %)	3 (14 %)	4 (19 %)	13 (62 %)	21

## Discussion

As a general rule, mucinous carcinomas represent a small fraction of neoplasms in different organs with about 3-10 % among ovarian neoplasms, 7 % of all colorectal cancers, 1.5 % of gastric carcinomas, and very rarely in the lung [12, 20]. Compared with conventional adenocarcinomas, mucinous tumors tend to be associated with young age, advanced tumor stage, and distinct molecular patterns, such as microsatellite instability and mutations of the *BRAF and KRAS* genes [21, 22].

Differentiation of primary ovarian mucinous carcinoma from metastases of other organs mostly appendix and colorectum can be challenging [13, 23]. This differentiation is pivotal and of clinical significance given that treatment protocols tend to be tailored to each organ site. For example platinum-based taxane agents are used in the treatment of primary ovarian carcinomas in contrast to fluorouracil which is the main chemotherapeutic agent used for metastatic carcinoma at the ovary originating from the colorectum [24, 25].

For a tumor to be considered mucinous carcinoma rather than a conventional adenocarcinoma depends on organ site and classification systems. Ovarian mucinous carcinomas show conspicuous amounts of intracellular mucin in more than 90 % of tumor cells [11]. In contrast, colorectal mucinous carcinoma is defined if at least 50 % of the tumor’s volume is composed of extracellular mucin [26]. Mucinous carcinomas are distinct from conventional and signet ring carcinomas in different organs with specific molecular alterations and clinical outcomes [25]. Common features between ovarian and colorectal mucinous carcinomas include higher prevalence at younger age and larger tumor size at presentation. In contrast, a higher proportion of ovarian mucinous carcinomas compared with serous carcinomas are diagnosed at a low stage, while colorectal mucinous carcinomas seem equally likely as non-mucinous carcinomas to present at a low stage [23, 27, 28].

More than 2 decades ago, Lightman et al. [7] reported low serum and tumor tissue zinc in patients with “malignant ovarian tumors”. However, in this publication, no mention of which kind of ovarian tumors were included in the study and not much clinical information was provided [7]. Relatively more recently, Bae et al. [8] reported that treatment of OVCAR-3 (malignant ovarian cell line) and NOSE (normal ovarian cell line) with zinc–citrate compound lead to induced zinc accumulation more in the former cell line than the latter. This resulted in a decrease in cell number and activity of M-aconitase in OVCAR-3 with increased apoptosis. The authors concluded that exposure to high concentration of zinc prevents the proliferation of OVCAR-3 cells [8]. The OVCAR-3 cell line used by Bae et al. is a human epithelial ovarian cancer cell line which was established from the malignant ascites of a patient with poorly differentiated “papillary adenocarcinoma” rather than mucinous carcinoma of the ovary [8, 9]. More mechanistic studies preferably with mucinous ovarian carcinoma cell lines e.g., RMUG-L, RMUG-S, MN-1, OMC-1 and MCAS [29] are recommended to further explore the role of zinc and zinc transporters in the pathogenesis and at least theoretically, influence management of mucinous carcinomas.

We and others conclusively established that zinc levels and hZip1 transporter are significantly decreased in prostate adenocarcinomas. The background and supporting evidence are fully and extensively presented in several reviews and reports [1–4]. This encouraged us to extend the study in the challenging mucinous carcinomas in different organs and to compare the hZip1 expression in mucinous versus non mucinous carcinomas in some of these organs.

Our novel finding in this report is that hZip1 showed consistently low expression in mucinous carcinomas of the ovary, colon, stomach and lung with no association between clinical stage and hZip1 scores in any of the mucinous tumors. Interestingly, we identified low expression of hZip1 in mucinous carcinomas versus non mucinous ones in three organs we performed the comparison in, namely; ovary, colon and stomach.

Specifically, hZip1 is strongly expressed in all cases of normal ovarian tissue and was moderately to strongly positive in 91 % of serous carcinomas. In contrast, 60 % of ovarian mucinous carcinomas were negative to weak positive for hZip1 expression. The second interesting finding is that there was a statistically significant lower expression of hZip1 in low grade ovarian serous compared to high grade serous carcinoma. Although it was not statistically significant, still hZip1 expression was lower in low grade compared to high grade mucinous carcinomas. These observations may indicate that the higher grade ovarian tumors may acquire different mechanisms to accumulate more zinc by switching off the inhibitory mechanisms which down express the zinc transporter *hZIP1* gene, or perhaps other members of the zinc transporter gene family.

The scenario was the same in colonic and gastric carcinomas with low expression of hZip1 in mucinous carcinoma versus high expression in conventional adenocarcinomas in those two organs. In the colon and stomach, 55 % and 22 % of mucinous carcinomas were negative to weakly positive for hZip1, respectively. In contrast, hZip1 expression was moderately to strongly positive in 96 % and 100 % of conventional adenocarcinomas, respectively in the colon and stomach. The number of conventional gastric adenocarcinomas included in the TMA was small (n = 45). In contrast to ovarian carcinomas, hZip1 expression was decreased as a function of increasing grade in both mucinous and non-mucinous tumors of the colon and stomach. This observation may point to different mechanisms involved in tumor progression regarding regulation of zinc concentration which need more mechanistic studies. In the lung, we also identified low expression of hZip1 in pulmonary mucinous carcinomas. Unfortunately, and due to un-availability of conventional lung adenocarcinoma TMA slides, we did not compare the expression of hZip1 in mucinous and conventional pulmonary carcinomas.

## Conclusions

hZip1 expression appears to be consistently decreased in mucinous carcinomas from a variety of organs relative to non-neoplastic tissues in those organs. However, hZip1 is also decreased as a function of increasing grade in both mucinous and non-mucinous tumors in the colon and stomach. These findings suggest that hZip1 plays a fundamental role in the carcinogenesis of some organs that is particularly accentuated in mucinous tumors. Additional studies are required to precisely define the nature of this role. However, practically, there is little value in low expression of hZip1 for differential diagnosis of mucinous carcinomas.

## Methods

### Tissue microarrays (TMAs)

All procedures were performed in compliance with relevant laws and institutional guidelines with approval of institutional review board. TMA slides purchased from US Biomax (Rockville, MD) were used in the present study. Ovary TMA slide comprised of 35, 152, 11 and 6 interpretable mucinous carcinomas, ovarian serous carcinomas, cancer adjacent normal tissue and normal (non-malignant) tissues, respectively in a single tissue core per case. It is not known if the mucinous carcinomas included in the TMA of the ovarian tumors are of primary ovarian origin or metastatic tumors. Colon TMA slide comprised of 51, 129 and 12 interpretable mucinous carcinomas, conventional adenocarcinomas and normal tissue, respectively in a single tissue core per case. Stomach TMA slide comprised of 32 interpretable mucinous carcinomas, 4 conventional adenocarcinomas and 3 cases of non-malignant normal and 2 tumor adjacent normal gastric tissue in duplicated cores per case. Lung TMA slide comprised of 21 interpretable mucinous carcinomas in a single tissue core per case.

### Immunohistochemistry (IHC)

hZip1 protein expression was determined by IHC using an anti-hZip1 antibody by standard protocol [1, 4]. Briefly, the TMA slides were deparaffinized in xylene and rehydrated by incubation in decreasing concentrations of ethanol. Antigen retrieval was done by heating in 10 mM Tris-EDTA (10 mM Tris base, 1 mM EDTA,, 0.05 % tween 20, pH9.0) buffer at 98 °C for 20 min, IHC staining was carried out by incubating slides in 5 % BlokHen (AvasLabs, Tigard, Oregon) followed by incubation overnight at 4 °C with hZip1 antibody in 5 % BlokHen followed by incubation with Horseradish peroxidase-labeled goat anti chicken IgY secondary antibody in a dilution of 1:200 (AvesLabs, Tigard, Oregon). Color was developed with DAB+ chromogenic substrate and hematoxylin was used as counterstain.

All sections were examined with an Olympus (BX53) microscope. The pictures were processed with cellSens Standard XV Image Processing software (Olympus Corporation of the Americas, Center Valley, PA). IHC scoring was performed by a board certified anatomic pathologist (MMD). The appearance of membrane and cytoplasmic associated hZip1 immuno-positivity of the glandular epithelial cells was used for scoring as previously described [1, 4]. Tumor staining intensity was semiquantitatively scored as negative (no staining), weak (1+) (<10 %), moderate (2+) (10-50 %) and strong (3+) (>50 %) positive depending on the percentage of positive cells.

### Statistical analysis

The mean scores of hZip1 expression in different categories were analyzed by the Student’s *t*-Test. The IHC scores were considered nominal to find out significance. *P* ≤ 0.05 was considered significant.

## Abbreviations

IHC: Immunohistochemistry
TMA: Tissue microarray
hZip1: Human zinc transporter member 1
